# Development of an inflammatory bowel disease (IBD) Patient‐Reported Experience Measure (PREM): A patient‐led consensus work and ‘think aloud’ study for a quality improvement programme

**DOI:** 10.1111/hex.13647

**Published:** 2022-11-06

**Authors:** Elena M. Sheldon, George Lillington, Kati Simpson, Kirsty Gibson, Lucy Chambers, Manfredi D'Afflitto, Nancy Greig, Theresa Stearn, Daniel Hind, Rachel Ainley, Gemma Winsor, Katie Ridsdale, Nikki Totton, Alan Lobo

**Affiliations:** ^1^ Sheffield Health and Related Research The University of Sheffield Sheffield UK; ^2^ Crohn's & Colitis UK Hatfield UK; ^3^ Gastroenterology and Liver Unit, Royal Hallamshire Hospital Sheffield Teaching Hospitals NHS Foundation Trust Sheffield UK

**Keywords:** inflammatory bowel disease, patient experience, quality improvement

## Abstract

**Background:**

Patient‐Reported Experience Measures (PREMs) are key in improving healthcare quality, but no PREM exists for inflammatory bowel disease (IBD). This study aimed to co‐produce a PREM with IBD service users for IBD service evaluation and quality improvement programme.

**Methods:**

A pool of 75 items was drawn from published survey instruments covering interactions with services and aspects of living with IBD. In Stage 1, during two workshops, eight expert service users reduced candidate items through a ranked‐choice voting exercise and suggested further items. During Stage 2, 18 previously uninvolved people with IBD assessed the face and content validity of the candidate items in ‘Think Aloud’ interviews. During two final workshops (Stage 3), the expert service users removed, modified and added items based on the interview findings to produce a final version of the PREM.

**Results:**

Stage 1 generated a draft working PREM mapped to the following four domains: Patient‐Centred Care; Quality; Accessibility; Communication and Involvement. The PREM included a set of nine items created by the expert group which shifted the emphasis from ‘self‐management’ to ‘living with IBD’. Stage 2 interviews showed that comprehension of the PREM was very good, although there were concerns about the wording, IBD‐relevance and ambiguity of some items. During the final two workshops in Stage 3, the expert service users removed 7 items, modified 15 items and added seven new ones based on the interview findings, resulting in a 38‐item PREM.

**Conclusions:**

This study demonstrates how extensive service user involvement can inform PREM development.

**Patient or Public Contribution:**

Patients were involved as active members of the research team and as research participants to co‐produce and validate a PREM for IBD services. In Stage 1, eight expert service users (‘the expert group’) reduced candidate items for the PREM through a voting exercise and suggested new items. During Stage 2, 18 previously uninvolved people with IBD (the ‘think aloud’ *participants*) assessed the validity of the candidate items in ‘Think Aloud’ interviews as research participants. In Stage 3, *the expert group* removed, changed and added items based on the interview findings to produce a final version of the 38‐item PREM. This study shows how service user involvement can meaningfully inform PREM development.

## 1 BACKGROUND

Crohn's disease (CD) and ulcerative colitis (UC), the main forms of inflammatory bowel disease (IBD), are lifelong debilitating conditions. Symptoms often follow an unpredictable trajectory between active disease and remission, which significantly affects the quality of life and psychosocial functioning.^1^ People living with IBD have heterogeneous needs which are often unmet by healthcare services. The views of healthcare professionals and patients differ concerning care priorities and quality.^2^ In 2021, IBD UK published a UK‐wide survey of 10,222 people with IBD, in which 28% rated their quality of care as only fair or poor.^3^ The report identified four areas for change: improvements in diagnosis and information provision; personalized care and support for self‐management; faster access to specialist advice and treatment and effective multidisciplinary team working.

Many IBD quality improvement initiatives take it for granted that organizations must learn from patients.^4^ Self‐report survey instruments are increasingly used as quality indicators,^5^ including in IBD,^6^ but not all measures are considered useful or effective.^7^ Patient satisfaction measures, which capture whether a patient received care that met their expectations are biased by previous experiences.^8^ Patient expectations are influenced by health status, frequency of service interaction and level of dependency on healthcare providers.^7^, ^9^ Satisfaction measures lack sensitivity, fail to distinguish between good and bad care and often overrate satisfaction due to gratitude bias.^10^ Simply put, high self‐reported satisfaction may not correlate with a positive healthcare experience.^9^ Patient‐Reported Experience Measures (PREMs) represent healthcare quality more accurately.^11^ PREMs capture ‘what’ happened in the care process, ‘how’ and ‘how often’.^8^, ^12^ Aspects of patient experience can become targets for service development,^5^ and are essential to quality improvement as they provide actionable data based on what matters to patients.^5^, ^7^, ^8^, ^9^, ^12^, ^13^

Survey instrument development requires a conceptual framework—a set of interlinked ideas that provide an understanding of, or are used to represent a phenomenon.^14^, ^15^ Thematic analysis of PREMs from a recently published conceptual framework maps out eight domains of patient experience of services: patient‐centred care; quality; integration; accessibility; involvement; communication; discomfort and environment and facilities.^16^ These domains strongly align with NHS England, National Clinical Guidelines Centre (NICE) and Institute of Medicine (IOM) definitions of quality in health care, which advocate that care should be patient‐centric, safe, effective, efficient and equitable.^17^, ^18^ A scoping review identified a range of IBD‐specific instruments that measure experience‐related concepts, such as patient satisfaction,^6^ patient knowledge,^19^ patient concerns,^20^ self‐efficacy^21^ and quality of care,^22^ but no validated PREM. To fill this knowledge gap, this study aimed to develop a PREM for people with IBDs to support IBD service evaluation.

Patient involvement in the development of survey instruments is recommended by regulators^23^; however, it is rarely well‐evidenced, except in a ‘cursory and poorly reported’ fashion,^24^ leading to differences in the understanding of survey items.^25^ Patient‐led approaches make the instrument development process more accountable and ensure that instruments are relevant, transparent and less subject to ambiguity.^26^ We combined patient leadership and qualitative research to ensure patients felt the PREM covered the most important issues (content validity)^27^ with a meaningful relationship between the items and what matters to them (face validity).^28^

The PREM was intended for use in a service evaluation alongside the Patient‐Activation Measure (PAM) of knowledge, skills and confidence in self‐management.^29^ Expert patients expressed concern that—for newly diagnosed patients, those on surgical pathways and those in a flare—some of the PAM's items inappropriately implied that disease management was wholly the patient's responsibility. Their response echoed the previous research^30^ and policy^31^ flagging that some conditions and cases require higher proportions of professional care to self‐management, that self‐management should be a choice and that poor self‐management often arises from low health literacy or overwhelming circumstances. Consequently, *the expert group* developed items which referred to behavioural determinants of ‘living well with IBD’ instead of ‘self‐management’. This broadened the instrument's scope to experiences beyond interactions with services. For this reason, in addition to Bull's experience framework,^8^, ^16^ we guided questionnaire development using two related conceptual frameworks for understanding how patient experiences might illuminate problems involving behavioural determinants. The COM‐B system—which understands behaviour as determined by capability, opportunity and motivation—is a synthesis of 19 behaviour change frameworks,^32^ the Theoretical Domains Framework (TDF), a synthesis of 33 theories of behaviour and behaviour change.^33^ Its developers describe the TDF as ‘an elaboration of the COM‐B model’ with ‘domains of theoretical constructs that map onto the COM‐B components and allow for a more detailed understanding of behaviour’.^34^ In line with the behaviour change wheel system, we use the COM‐B model to talk in broad terms, and the TDF to talk in more narrow terms about behavioural determinants addressed by different PREM items. The COM‐B and TDF are relevant because a large part of the experience of living with IBD involves the adoption and maintenance of what clinical academics would call ‘self‐management’ behaviours,^19^, ^20^, ^21^ although this term is not preferred by *the expert group*, and the TDF is often used to identify barriers to, and facilitators of, desirable self‐management behaviour.^35^ Our scoping review indicated that 339 items on 20 existing IBD measurement instruments were not symptom measures, nor were they measured constructs to do with capability (*n* = 213 items), opportunity (*n* = 99) and motivation (*n* = 87). The TDF includes a wider range of determinants for successful self‐management than the PAM and is often used to identify targets for the improvement of supportive services.^36^ Both patients and clinicians have therefore recognized the utility of the COM‐B and TDF for areas of living well with IBD where the patient can have more agency in managing the condition.

## 2 METHODS

### 2.1 Overview

Service users led the development and validation of the PREM across a three‐stage process: Stage 1—theme selection and item generation; Stage 2—face and content validity testing and Stage 3—item reduction and scale generation. A group of seven expert service users (*the* ‘expert group’) led Stages 1 and 3, supported by a *project team* (E. M. S., D. H., A. L.), partners at Crohn's & Colitis UK (R. A., G. W.) and a statistician (N. T.). Crohn's & Colitis UK selected *the expert group* of seven people with IBD (co‐authors G. L., K. S., K. G., L. C., M. D., N. G., T. S.) from a range of professional backgrounds using online methods. *The expert group* were recruited via the Crohn's & Colitis UK website, social media and with key contacts who had relevant disease experience using a REC‐approved advert. The group were selected based on their previous research experience, professional background and a range of geographic locations. This included, but is not limited to, an editor in survey research; a Crohn's & Colitis UK Health Service Project Manager for Scotland; an IBD UK patient representative; a medical student; a self‐employed Organisational Development Coach, Facilitator and Leader and a lay member of the Research Strategy and Funding Committee for Crohn's and Colitis UK. All individuals were known to Crohn's & Colitis UK as having experience in IBD advocacy and had previous voluntary work experience, for example in the readers' panel, implementing self‐management projects or as lay members of the charity's committees. Successful applicants were contacted via email to make introductions to *the project team*. Informed consent was received to record all workshops.

In Stage 2, the *project team* interviewed other service users (‘the think‐aloud group’) as research participants to test the face and content validity of the instrument with independent patients.

### 2.2 Stage 1: Theme selection and item generation

*The project team* identified domains of the patient experience from three sources: (1) PREMs from analogous contexts^8^; (2) survey findings, policy documents and IBD UK standards identified by a Crohn's & Colitis UK exercise summarizing what matters to people with IBD and (3) principles for patient‐centred care.^11^ *The expert group* considered the appropriateness of the following patient experience domains at an online workshop: patient‐centred care; quality; integration; accessibility; involvement; communication; discomfort and environment and facilities.^16^ These candidate items and domains were used only as stimuli for discussion.

*The expert group* recommended the inclusion of items in the PREM about determinants (barriers and facilitators) of service user behaviour. *The expert group* did not have to accept any item, its wording, or any theme. In the event, through discussion amongst themselves, patients re‐categorized items in domains most meaningful to them. The academic frameworks were retained to allow for comparison with patient‐derived themes and their own ‘second‐degree’ constructs.^37^ Based on individuals' availability, four *expert group* members participated in individual one‐to‐one sessions with the project team to adapt candidate PREM items or create new ones that mapped to the TDF,^33^ ensuring relevance to their lived experience while avoiding implications that barriers derived from the patient rather than the service (see Supporting Information: 1). An applied health service researcher with experience in using the TDF (D. H.) and a graduate psychologist (E. M. S.) trained and assisted the four *expert group* members with this mapping exercise. New items mapped to the following TDF domains: Knowledge, Skills and Memory (Psychological Capability); Social Role and Identity; Beliefs about Consequences, and Goals (Reflective Motivation); Emotion (Automatic Motivation) and Social influences and Support (Social Opportunity). *The project team* developed sub‐themes, both positive and negative, for each TDF domain, generating a pool of 75 candidate items that reflected good or poor patient experience.

After the initial meeting, *the expert group* completed a ranked‐choice voting exercise by allocating points to each of the eight experience domains and 75 candidate items on a spreadsheet. The purpose of the ranking was to allow *the expert group* to anonymously choose candidate domains and items in order of preference, where those receiving the fewest or no votes were eliminated. Individuals from *the expert group* completed the voting spreadsheet independently and the project team collated the results to present at the subsequent workshop. Based on their experience, *the expert group* modified existing items or wrote new ones to reflect anything they considered imprecise, in error or absent. These items were mapped to the top five rated PREM domains: Patient‐Centred Care; Quality; Accessibility; Communication and Involvement. *The project team* presented a working PREM based on the results of the voting exercise and the new items based on the TDF. Items were added, improved or removed with reference to Streiner and Norman's criteria: too complex; ambiguous; double‐barrelled; jargon; value‐laden; negatively worded or too lengthy.^38^ *The expert group* chose a Likert scale ranging from ‘Not At All’ to ‘To a Very Large Extent’ and wrote a definition for how the term ‘Care Team’ should be used and understood. The co‐produced pool of candidate items was combined to represent a draft working PREM for use in Stage 2.

### 2.3 Stage 2: Face and content validity testing of shortlisted items with a ‘think aloud’ group

Stage 2 work was conducted by E. M. S. (BSc), a female psychologist with qualitative research experience. To understand face validity^28^ (whether items were acceptable to people with IBD), the study used the ‘think aloud’ protocol,^39^ in which participants were asked to say what came into their mind as they completed the survey instrument. A brief unstructured interview followed in which participants were asked to clarify any matters arising during the ‘think aloud’ interview and to evaluate content validity (the extent to which candidate items cover aspects of care that are important to people with IBD).^27^ This included items about how participants found the overall length of the questionnaire, the Likert scale and general formatting. Crohn's & Colitis UK identified a purposive sample of previously uninvolved people with IBD via social media and the charity's website using REC‐approved standard advertisement text, inviting patients to opt‐in by email. Eligible participants were adults (aged 16 years or over) with IBD (CD or UC) and the capacity to give fully informed consent. Interviews of 30–40 min were conducted by telephone or videoconference. Participants were provided with a £20 shopping voucher as compensation for their time.

Interview transcripts from encrypted recordings were analysed by E. M. S., D. H. and K. R., in NVivo (QSR International) version 12, using the National Centre for Social Research ‘Framework’ qualitative data analysis method, which involves five stages: familiarization, identifying a thematic framework, indexing, mapping and interpretation.^40^ Following Morgan, we understand codes as a system for marking up ‘parts of the text that are of special interest’ and themes as converting ‘codes into core concepts that represent the most important aspects of the results’.^41^ In this case, the aim of the study is to ensure that items on the final PREM would reflect the range and content of deductively and inductively derived themes. Our analysis of think‐aloud data combined deductive coding (based on Streiner and Norman's criteria^38^ for face validity and the conceptual frameworks^8^, ^16^, ^32^, ^33^ for content validity) with the inductive development of codes for ‘parts of the text of special interest’,^41^ with content not already covered by the frameworks. In general, these new inductively derived codes were developed during the closing brief unstructured interview (see above) and involved IBD context‐specific responses to the face validity of items drawn from other survey instruments. For instance, interviewees felt that some questions presupposed a more predictable disease course than was typical with IBD.

Following Francis et al.,^42^ we specified a priori that 12 interviews would be considered analysed before considering saturation, allowing for stopping after every two further interviews if two coders agreed that no new themes were identified. Interviewing ran ahead of analysis which, retrospectively, showed that data saturation was achieved in the first 14 interviews with no substantial different suggestions for question modification or new items thereafter. The final four interviews were included and the sample size (*n* = 18) was in line with methodological research that shows that 9–17 interviews are generally sufficient for saturation with a fairly homogenous study sample and narrowly defined items.^42^, ^43^

### 2.4 Stage 3: Item reduction and scale generation

Interview findings from Stage 2 were summarised and presented to *the expert* group during two online workshops (see Supporting Information: 7 and 8 for the workshop slides). The *expert group* assessed the importance of each interview finding, agreed on the formatting of the PREM, including item order, and considered new items for inclusion. Where conflicting views between ‘think aloud’ *participants* and *the expert group* were identified, *the project team* proposed different solutions for each item. Where verbal agreement was not reached during the workshop, *the expert group* independently voted ad hoc by email on their preferred solution and suggested new items where required by interview findings (Supporting Information: 9). Gunning Fog index scores^44^ operationalized Streiner and Norman's ‘readability’ criterion.^38^ Items with scores of nine or over were rewritten where possible. The Gunning Fog index is widely used in health research^45^ and provides an easily available, free‐to‐use web tool which *the project team* and *expert patient group* used to experiment with alternative wordings and sentence lengths. The ideal score for readability with the Fog index is 7 or 8; which is the equivalent of Years 8‐9 in the UK schooling system and the seventh and eighth grades in the US education system.^44^ The project team allowed scores of 9 with exceptions that allowed for contextually specific words with which patients were likely to be comfortable, for example ‘colitis’ and ‘hospital’. At the final workshop, voting exercise results and revisions were presented. Outstanding issues, for example, where items received no majority vote, were resolved through discussion.

## 3 RESULTS

### 3.1 Stage 1: Theme selection and item generation

Of 75 candidate items in the ranked‐choice voting exercise, 36 were selected for inclusion, along with three new items (Supporting Information: 2): ‘I know how to contact the Care Team between appointments if I need to’; ‘It is easy to get the help I need from a member of the Care Team when I need it’ and ‘I feel able to discuss my mental health with the Care Team if I want to’. In one‐to‐one sessions, *the expert group* added 14 self‐management‐based items, to replace the PAM‐13. After the addition, removal or modification of items at the second workshop, a 35‐item survey draft instrument was developed for use in Stage 2.

### 3.2 Stage 2: Face and content validity

Eighteen participants took part in ‘think aloud’ interviews, with a median age of 32 (range 26–82) years. Demographics are shown in Table 1. The average length of interviews was 40 min, with a range of 46 min. The shortest interview was 17 min and 47 s; the longest was 1 h and 3 min. Participants highlighted problems with 10 items (Table 2), including items that were too ambiguous, too value‐laden, contained jargon or were negatively worded. This later resulted in the removal of two items and the rewording of five items by the expert group.

**Table 1:** Respondent demographics for PREM Stage 2 ‘think aloud’ interviews (*n* = 18)

Characteristic	Number of respondents (%)
Gender	
Female	12 (66.7)
Male	6 (33.3)
Age	
20–29	4 (22.2)
30–39	9 (50)
40–49	3 (16.7)
50–59	0 (0)
60–69	1 (5.6)
70–79	0 (0)
80–89	1 (5.6)
IBD diagnosis	
Crohn's	14 (77.8)
Ulcerative colitis	4 (22.2)
Ethnicity	
White British	16 (88.9)
Tamil	1 (5.6)
Black Caribbean/British	1 (5.6)
Employment status	
Full‐time	9 (50)
Part‐time	4 (22.2)
Self‐employed	3 (16.7)
Retired	2 (11.1)
Education level	
Postgraduate degree	5 (27.8)
Degree	9 (50)
Secondary education	4 (22.2)
Region in England	
Yorkshire and the Humber	9 (50)
South East	3 (16.7)
North East	2 (11.1)
South Central	1 (5.6)
London	1 (5.6)
South West	1 (5.6)
East Midlands	1 (5.6)

Abbreviations: IBD, inflammatory bowel disease; PREM, Patient‐Reported Experience Measure.

**Table 2:** Interview participant quotes about items in the Stage 2 version of the PREM

Streiner and Norman criteria	Item	Quote	Final outcome for item
Ambiguity	The Care Team knows how I feel emotionally while they are treating me	Well I've never had an emotional sort of complaint talking with the Care Team (Participant 14)	Reword item
	I feel that I have the emotional strength to live with IBD on a day‐to‐day basis	Day to day [I'm] not sure about that, it depends on what day of the week it is (Participant 2)	Reword item
	My mental health and well‐being affects my ability to live with Crohn's or Colitis	It just seems a bit open ended […] I don't really know where you're driving on that one (Participant 6)	Remove item
Value‐laden terms	I am able to access sufficient support from the wider IBD community to help me live with Crohn's or Colitis	I felt like it was saying I should be active in the IBD community, putting a little bit of pressure on, when that's not for me, I've tried it and didn't want that contact reminder. (Participant 17)	Reword item
	My Care Team understands what's important to me as an individual (my preferences and priorities in healthcare and beyond)	…but I don't think that's a bad thing. I think they're focused on my disease as they should be as they're experts and they want to get some treatment going to make you feel better, and whatever's important to me in my life doesn't really matter	Reworded
Jargon	I believe that my care and treatment plan will have beneficial effects	Well how do I know that, because I'm not a medical practitioner? So I can only relate that to how I feel, I guess, and my hope (Participant 13)	Reword item
Negatively worded items	My mental health and well‐being affect my ability to live with my Crohn's or Colitis	It was at the wrong end of the scale, you expect the 5 s to be the positives and the 1 s to be the negatives, whereas that one was switched round (Participant 1)	Remove item

Abbreviations: IBD, inflammatory bowel disease; PREM, Patient‐Reported Experience Measure.

Some items had few objections, particularly those related to mental health and other nonmedical aspects of living with IBD. Participants liked that the PREM covered broader aspects of living with a chronic condition, which is often neglected in clinical encounters.

The length of the PREM was acceptable to participants and most items were considered clear, relevant and easy to understand. Some participants suggested that the term ‘Care Team’ could be confusing, given the multidisciplinary nature of IBD care. For instance, while some service users are on a surgical pathway and regularly interact with surgeons, dieticians and gastroenterologists, others who are in remission might only see the IBD nurse specialist on an annual basis. Participants recommended removing items where they perceived overlap or repetition, and suggested 11 new items based on aspects of their experience that they felt were missing.

Participants described some items as inappropriate because of the unpredictability of IBD. With reference to Item 34 (‘I have a clear picture of where I want to be in terms of my Crohn's or Colitis’), one participant (P04) explained: ‘You just never know because all of a sudden you can have a flare out of absolutely nowhere so it's hard to have a clear picture. … I don't think a Crohn's or Colitis journey is a clear one for anyone’.

Some items were considered ambiguous, for example, whether mental well‐being items referred to mental health conditions or the impact of living with IBD, as well as difficulties with how emotions fluctuate alongside symptom severity:

With diseases like Crohn's and Colitis, because it can go up and down so much, the ebb and flow of that changes the other stuff around it. […] I definitely know my emotions and mental health change depending on the activity of my disease. (P18)

### 3.3 Stage 3: Item reduction and scale generation

**Figure 1:**
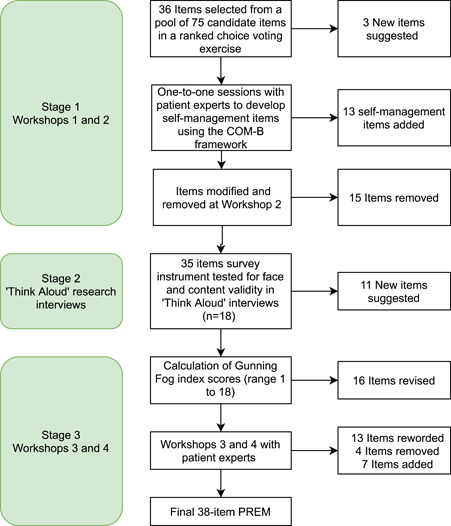
PREM development process. PREM, Patient‐Reported Experience Measure.

Initial Gunning Fog index scores ranged from 1 to 18 (median 10; see Supporting Information: 3). Sixteen items were revised to improve readability (recalculated score range 11; median 8), allowing for three‐syllable words with which IBD service users are familiar, such as ‘hospital’. For example, ‘My Care Team understands what's important to me as an individual (my preferences and priorities in healthcare and beyond’ was reworded to ‘The Care Team understands what matters to me (in healthcare and beyond)’, reducing the Gunning Fog index score from 18 to 8. At the third workshop, based on the interview findings and Gunning Fog index scores, *the expert group* removed three and reworded seven items (Supporting Information: 4). The Likert scale, layout and item order were finalized. *The expert group* voted to reword five, remove one and include seven new items suggested by the ‘think aloud’ *participants* (Supporting Information: 5). *The expert group* reworded each of the included items, resulting in the final 38‐item PREM (Figure 1). The length of the PREM was deemed appropriate by *the expert group*. The PREM was re‐structured by *the expert group* using the following three headings: ‘The Care Team’; ‘What Matters to Me’ and ‘Living with Crohn's and Colitis’. Supporting Information: 6 shows how individual items map to the conceptual frameworks which informed the PREM's development. To ensure relevance to decision‐makers, we mapped 27 of these items to policy imperatives from the IBD UK standards (Table 3; see Section 4). For example, item 38 ‘I have a personalised written care plan’ was mapped to Statement 7.1 (‘A personalised care plan should be in place for every IBD patient, with access to an IBD nurse specialist and telephone/email advice line’) as per the IBD UK standards.

**Table 3:** PREM mapped to IBD UK standards where applicable

Item no.	AWARE‐IBD PREM	IBD standards
*The Care Team*		
1	I know who the different people are in the Care Team looking after me	**Statement 1.1** Patients should be cared for by a defined IBD multidisciplinary team led by a named consultant adult or paediatric gastroenterologist. **Statement 1.9** Clear information about IBD, the local IBD service and patient organizations should be accessible in outpatient clinics, wards, endoscopy and day‐care areas
2	I know how to contact the Care Team between appointments if I need to	**Statement 2.4** All patients should be provided with a point of contact and clear information about pathways and timescales while awaiting the outcome of tests and investigations. **Statement 4.2** All patients with IBD should be provided with clear information to support self‐management and early intervention in the case of a flare. **Statement 7.1** A personalized care plan should be in place for every IBD patient, with access to an IBD nurse specialist and telephone/email advice line.
3	I can get a response from the Care Team by the end of the next working day when experiencing a flare	**Statement 4.3** Rapid access to specialist advice should be available to patients to guide early flare intervention, including access to a telephone/email advice line with response by the end of the next working day.
4	I feel that the Care Team has enough time for me when I talk to them	
*What Matters to Me*		
5	I know the person on the Care Team who coordinates my care	**Statement 1.1** Patients should be cared for by a defined IBD multidisciplinary team led by a named consultant adult or paediatric gastroenterologist. **Statement 1.9** Clear information about IBD, the local IBD service and patient organizations should be accessible in outpatient clinics, wards, endoscopy and day‐care areas. **Statement 7.1** A personalized care plan should be in place for every IBD patient, with access to an IBD nurse specialist and telephone/email advice line.
6	The Care Team understands the impact my Crohn's or Colitis has on my life	**Statement 3.2** After diagnosis, all patients should have full assessment of their disease, nutritional status, bone health and mental health, with baseline infection screen, to develop a personalized care plan. **Statement 3.3** Patients should be supported to make informed, shared decisions about their treatment and care to ensure these take their preferences and goals fully into account.
7	My concerns are taken seriously when I talk to the Care Team	
8	The Care Team ask how I feel while they are treating me	
9	I feel I can approach the Care Team to discuss any concerns about my treatment and its effects on my life	**Statement 3.3** Patients should be supported to make informed, shared decisions about their treatment and care to ensure these take their preferences and goals fully into account. **Statement 5.4** Patients with IBD being considered for surgery should be provided with information in a format and language they can easily understand to support shared decision‐making and informed consent and offered psychological support. **Statement 7.1** A personalized care plan should be in place for every IBD patient, with access to an IBD nurse specialist and telephone/email advice line.
10	The Care Team understands what matters to me (in healthcare and beyond)	**Statement 7.1** A personalized care plan should be in place for every IBD patient, with access to an IBD nurse specialist and telephone/email advice line.
11	I have the confidence to express my needs and concerns with the Care Team	
12	I feel that the Care Team do their best to give me the care I need	**Statement 1.2** Multidisciplinary team meetings should take place regularly to discuss appropriate patients.
13	I am involved in decisions about my care and treatment	**Statement 3.3** Patients should be supported to make informed, shared decisions about their treatment and care to ensure these take their preferences and goals fully into account. **Statement 7.1** A personalized care plan should be in place for every IBD patient, with access to an IBD nurse specialist and telephone/email advice line
14	I feel I have a good relationship with my Care Team	
15	I am treated with dignity and respect by the Care Team	
16	I usually see the same person from the Care Team at each appointment (either face‐to‐face, telephone or online).	
17	The Care Team offers me appointments in a format that suits me, such as face to face, by telephone or video call	
18	There is good coordination between the different people involved in my care and treatment: (1) Within my Care Team (e.g., doctors, IBD nurse specialists, surgeons, dietitians)	**Statement 5.1** Patients should have access to coordinated surgical and medical clinical expertise, including regular combined or parallel clinics with a specialist colorectal surgeon (paediatric colorectal surgeon where appropriate) and IBD gastroenterologist. **Statement 1.2** Multidisciplinary team meetings should take place regularly to discuss appropriate patients.
	(2) Between my Care Team and other teams in the hospital that I may be in contact with (e.g., rheumatology, dermatology, obstetrics)	**Statement 1.1** Patients should be cared for by a defined IBD multidisciplinary team led by a named consultant adult or paediatric gastroenterologist.
	(3) Between my Care Team and my GP Practice	**Statement 3.6** GPs should be informed of new diagnoses and the care plan that has been agreed within 48 h. **Statement 7.3** Clear protocols should be in place for the supply, monitoring and review of medication across primary and secondary care settings. **Statement 7.5** Any reviews and changes of treatment in primary or secondary care should be clearly recorded and communicated to all relevant parties within 48 h.
	(4) Between my Care Team and other healthcare professionals	
19	The Care Team will refer me to other services if needed (e.g., mental health services)	**Statement 3.2** After diagnosis, all patients should have full assessment of their disease, nutritional status, bone health and mental health, with baseline infection screen, to develop a personalized care plan. **Statement 5.7** Patients and parents/carers should be provided with information about postoperative care before discharge, including wound and stoma care, and offered psychological support. **Statement 6.8** On admission, patients with IBD should have an assessment of nutritional status, mental health and pain management using validated tools and be referred to services and support as appropriate.
20	In general, I am able to understand all the information the Care Team gives me	
21	Thinking about the last time I was given information by the Care Team about my care and treatment: (1) It was given in a way that was easy to understand (2) It met my needs (3) It was relevant to me and my needs (4) I had the opportunity to discuss and ask questions about it (5) I liked the way it was given (e.g., verbal or on paper)	**Statement 1.9** Clear information about IBD, the local IBD service and patient organizations should be accessible in outpatient clinics, wards, endoscopy and day care areas. **Statement 3.3** Patients should be supported to make informed, shared decisions about their treatment and care to ensure these take their preferences and goals fully into account. **Statement 7.1** A personalized care plan should be in place for every IBD patient, with access to an IBD nurse specialist and telephone/email advice line.
22	The Care Team has recommended or directed me to good, reliable information resources, such as charities and the NHS website	**Statement 3.5** Patients should be signposted to information and support from patient organizations. **Statement 7.2** Patients should be supported in self‐management, as appropriate, through referral or signposting to education, groups and support.
23	The Care Team informs me about opportunities to take part in research studies and clinical trials	**Statement 1.17** IBD services should encourage and facilitate involvement in multidisciplinary research through national or international IBD research projects and registries.
24	The frequency of my routine appointments is acceptable	**Statement 7.7** All IBD patients should be reviewed at agreed intervals by an appropriate healthcare professional and relevant disease information recorded. **Statement 7.8** A mechanism should be in place to ensure that colorectal cancer surveillance is carried out in line with national guidance and that patients and parents/carers are aware of the process.
25	I am able to easily access toilet facilities at the hospital	**Statement 6.2** Where en suite rooms are not available, inpatients with IBD should have a minimum of one easily accessible toilet per three beds on a ward.
26	I know how to provide feedback on the service, should I want to	**Statement 1.7** Patients and parents/carers should have a voice and direct involvement in the development of the service.
*Living with Crohn's or Colitis*		
27	I know what care and treatment options are available for my Crohn's or Colitis	**Statement 1.13** Patients should be fully informed about the benefits and risks of, and the alternatives to, immunomodulator and biological therapies, including surgery. **Statement 3.3** Patients should be supported to make informed, shared decisions about their treatment and care to ensure these take their preferences and goals fully into account.
28	I understand how Crohn's or Colitis affects me physically	**Statement 1.9** Clear information about IBD, the local IBD service and patient organizations should be accessible in outpatient clinics, wards, endoscopy and day care areas. **Statement 3.5** Patients should be signposted to information and support from patient organizations.
29	In general, I feel that I can mentally cope with my Crohn's or Colitis	
30	I feel able to discuss my mental health with the Care Team if I want to	
31	I *can do* all the tasks that my care team ask me to do at home (such as manage my diet, lifestyle, treatment)	**Statement 7.1** A personalized care plan should be in place for every IBD patient, with access to an IBD nurse specialist and telephone/email advice line. **Statement 7.2** Patients should be supported in self‐management, as appropriate, through referral or signposting to education, groups and support.
32	I *remember to do* all of the tasks that my care team ask me to do (such as take tablets, keep a food diary, etc.)	**Statement 7.1** A personalized care plan should be in place for every IBD patient, with access to an IBD nurse specialist and telephone/email advice line. **Statement 7.2** Patients should be supported in self‐management, as appropriate, through referral or signposting to education, groups and support.
33	I am able to keep track of my symptoms	**Statement 7.2** Patients should be supported in self‐management, as appropriate, through referral or signposting to education, groups and support.
34	I feel it is important to take an active role in my own healthcare	**Statement 7.2** Patients should be supported in self‐management, as appropriate, through referral or signposting to education, groups and support.
35	I get enough support from the people around me to help me live with Crohn's or Colitis (such as friends, family or people at work)	
36	I can access support from the IBD community to help me live with Crohn's or Colitis, if I want to (such as charities, online groups, support groups)	**Statement 1.9** Clear information about IBD, the local IBD service and patient organizations should be accessible in outpatient clinics, wards, endoscopy and day care areas. **Statement 7.1** A personalized care plan should be in place for every IBD patient, with access to an IBD nurse specialist and telephone/email advice line. **Statement 7.2** Patients should be supported in self‐management, as appropriate, through referral or signposting to education, groups and support.
37	I believe that my care and treatment will benefit me	**Statement 3.3** Patients should be supported to make informed, shared decisions about their treatment and care to ensure these take their preferences and goals fully into account.
38	I have a personalized written care plan	**Statement 7.1** A personalized care plan should be in place for every IBD patient, with access to an IBD nurse specialist and telephone/email advice line.

Abbreviations: IBD, inflammatory bowel disease; PREM, Patient‐Reported Experience Measure.

## 4 DISCUSSION

### 4.1 Principal findings

IBD service users developed a 38‐item survey instrument to capture the experience of healthcare delivery and living with IBD. They selected and rephrased items from other instruments and proposed new items based on their experience and on the interviews with their peers. Their feedback on the PAM‐13 resulted in the formulation of new items, based on a more robust framework, reflecting how people with IBD view and manage their condition. This rigorous patient‐led process should ensure the PREM's relevance, acceptability and validity and keep service users at the centre of an initiative designed to improve the person‐centredness of care.^46^ To our knowledge, this is the first and only PREM for IBD healthcare settings, based on Bull et al.s'^8^ definition—‘what’ happened during an episode of care from the patient perspective. Other IBD‐specific tools which discuss experience, such as the WE‐CARE IBD Score,^6^ contain Likert scales which focus on satisfaction rather than the extent to which a phenomenon occurred. The use of more than one conceptual framework is often relevant in complex situations, where a high‐level abstraction stands in for multiple entities that can be understood in multiple ways or approached with different interests and purposes in mind because frameworks never deal with phenomena in their entirety.^15^ Our PREM maps IBD‐specific experiences to valid constructs representing broader social scientific processes (the TDF), as well as policy imperatives (UK IBD standards) and broader constructs for understanding experience (Bull's framework). Mapping the PREM items to the TDF, IBD UK standards and Bull's framework invites other researchers to use the PREM in IBD quality improvement exercises where a health psychology perspective is desirable. As such, it has wide application in research and service improvement contexts.

Positive feedback from service users from different areas of the United Kingdom, and user‐testing with numbers adequate for saturation,^47^ provides confidence that this instrument has relevance and utility. Purposive sampling methods from social media and the internet contributed to the homogenous sample in this study in terms of age and ethnicity. Service users were all aged over 25 years, warranting investigation as to how developmentally appropriate^48^ its content and language are for younger adults. When translated from English, the cultural appropriateness of the wording and concepts should be assessed for similarity to the source language and how meaningful they are to the speakers in the target population. A further limitation is that the health literacy levels of the ‘think aloud’ *participants* were not assessed. Future research will assess different forms of reliability and validity in more representative quality improvement cohorts of people with IBD, and investigate the face and content validity of the instrument in young adults.

IBD UK standards provide a consensus of how high‐quality care is defined.^3^ We have related experience to such quality standards in the mapping exercise, with 28 of the PREM items defined by patients mapping to one or more standards. As such, the PREM can give a clear description, from a patient's perspective, of the extent to which they are actually experiencing these standards in their care. Services might use the responses as robust, patient‐reported evidence of meeting the quality standards. Ten items in the PREM are not represented in the IBD UK standards. These include items which cover important issues, including the ability of an individual to mentally cope with their IBD; that they are treated with dignity and respect; that they understand the information given to them; that their concerns are taken seriously; that they have the confidence to express their needs and that appointments are in a format that suits them. Future research and iterations of the IBD standards should consider whether such items should be included within the overall standards of care.

Positive patient experience is associated with higher levels of care quality and clinical effectiveness.^39^ Experience measures are increasingly used to complement process, clinical and cost data as evidence of a service's compliance with top‐down policy,^8^ and used as a bottom‐up method of identifying targets for improvement.^9^ As such, PREMs have the potential to benefit patients as well as to provide system‐wide benefits. However, clinical teams can find experience data removed from day‐to‐day concerns or difficult to translate into actionable improvements and the use of PREMs without structured staff training is not recommended.^5^

The AWARE‐IBD collaboration (doi:10.17605/OSF.IO/H7FCP) is currently collecting PREM data using a co‐produced web‐based application, allowing service‐user completion from home. The purpose of using the instrument is to make the patient experience visible to healthcare professionals so that they can optimize care at an individual and service level. Future evaluations will look at how PREM data are used to structure clinical encounters. PREM data will also be used in time series analyses to understand the success of patient‐led quality improvement efforts.

## 5 CONCLUSIONS

This paper describes a patient‐led process for the development and validation of a 38‐item IBD PREM. We are confident that our sample was adequate to explore content and face validity across two major subpopulations of IBD given the strength of complementary public involvement. However, further validation is required to test the psychometric properties of the PREM and to determine how patient experience data can evaluate the effects of changes in service delivery, particularly for underrepresented patient groups in IBD.

## AUTHOR CONTRIBUTIONS

*Design, conduct, analysis, interpretation and drafting of this article*: Elena M. Sheldon. *Design, analysis and interpretation for this article*: Daniel Hind, Katie Ridsdale, Nikki Totton and Alan Lobo. *Design, conduct and review this article*: Rachel Ainley and Gemma Winsor. *Conduct and interpret the study (as patient experts) and review the article*: George Lillington, Kati Simpson, Kirsty Gibson, Lucy Chambers, Manfredi D'Afflitto, Nancy Greig and Theresa Stearn. All authors read and approved the final manuscript.

## CONFLICT OF INTEREST

The authors declare no conflict of interest.

## ETHICS STATEMENT

London—Riverside Research Ethics Committee (REC) reference granted a favourable opinion (20/PR/0974). *The expert group members* were collaborators (not research participants), and worked in a service improvement (not research) paradigm, but gave informed consent for workshops to be recorded and preferred wording accurately captured.

## Supporting information

Supplementary information: Click here for additional data file.

## Data Availability

The data sets used and/or analysed during the current study are available from the corresponding author upon reasonable request.
