# Supplementary material for: Development of an inflammatory bowel disease (IBD) Patient‐Reported Experience Measure (PREM): A patient‐led consensus work and ‘think aloud’ study for a quality improvement programme
Source: Health Expect. 2022 Nov 6;26(1):213–25. doi: 10.1111/hex.13647 (PMC9854292; doi:10.1111/hex.13647)
Supplement: Supplementary file 1 — Supplementary information: [file HEX-26--s001.pdf]

**Supplementary Material 1. Items on determinants (barriers and facilitators) of desirable items, added through discussions in four 1:1 workshops with members of *the expert group*, to replace the Patient Activation Measure (PAM-13) survey.**

| **COM-B domain** | **TDF domain** | **Suggested questions** |
|---|---|---|
| Psychological Capability | Knowledge | I understand how Crohn's or Colitis is affecting my body |
| Psychological Capability | Knowledge | I know what care and treatment options are available for *my* Crohn's or Colitis (including any alternatives to my current treatment) |
| Psychological Capability | Skills | On an ongoing basis I am able to monitor my disease activity |
| Psychological Capability | Memory | I remember to do the tasks that my care team have asked me to do (e.g. take medication, keep a food diary, etc.) |
| Reflective Motivation | Social role and identity | I feel it is important to take an active role in my own healthcare |
| Reflective Motivation | Social role and identity | My Care Team understands what's important to me as an individual (my preferences and priorities in healthcare and beyond) |
| Reflective Motivation | Beliefs about capabilities | I have the confidence to express my personal needs and concerns with members of the Care Team |
| Reflective Motivation | Beliefs about capabilities | I can do the tasks that my care team have asked me to do at home (e.g. manage my diet, lifestyle changes, take medication) |
| Reflective Motivation | Beliefs about consequences | I believe that my care and treatment plan will have beneficial effects |
| Reflective Motivation | Goals | I have a clear picture of where I want to be in terms of my Crohn's or Colitis |
| Automatic Motivation | Emotion | My mental health and well-being affect my ability to live with my Crohn's or Colitis |
| Automatic Motivation | Emotion | I feel that I have the emotional strength to live with IBD on a day to day basis |

| | | |
|---|---|---|
| Social Opportunity | Social influences and support | I receive sufficient social support from the people around me to help me live with Crohn’s or Colitis |
| Social Opportunity | Social influences and support | I am able to access sufficient support from the wider IBD community to help me live with Crohn’s or Colitis |

**Supplementary Material 2. Total scores from *the expert group* ranking of Candidate Items (n=6), suggested wording by email, and agreed Item text in Workshop 2 (Stage 1).**

| Candidate Item | Total score | Suggested wording | Agreed Item |
|---|---|---|---|
| *Involved in decisions about care and treatment* | 290 | Reword as "I am involved in decisions about care and treatment" or “Was I given choices and were the consequences of each explained?” | *I am involved in decisions about my care and treatment* |
| *My care is well coordinated between my GP and Gastroenterologist (e.g. blood tests, monitoring, drug levels)* | 240 | Simplify to "my care is well coordinated" or reword as "My care is well co-ordinated between all of the medical professionals treating me" | **There is good coordination between the different health professionals involved in my care and treatment (e.g. my hospital Care Team, teams in other hospital departments, my GP, other health professionals)* |
| *Able to get help quickly when I have had a flare* | 240 | Reword to introduce a note of self-management "When I have a flare-up I am able to get help quickly, if I feel I need it" | *I am able to quickly contact different members of the Care Team when I need to* |
| *Given information at the time I needed it* | 230 | Reword as "I was given information at the time I needed it" or add "and sign-posted to patient-groups and organisations" | *About the information I was given: c) It was timely (relevant to my needs at the time I needed it)* |
| *Were you told what you thought (felt) was necessary about the results of tests and examinations while you were in the institution?* | 190 | Discussed ambiguity of question, need to make clear it is what the patient thinks necessary. Also whether to include ‘interventions’. | Not included |

| | | | |
|---|---|---|---|
| *Health team fully up to date with my situation* | 150 | | *The Care Team is fully up to date with my medical situation* |
| *Did you experience that the IBD Team had enough time for you when you needed it?* | 130 | Reword as "When meeting members of my health team they give me enough time to talk to them about what matters to me (agree/disagree scale)" | **I feel that the Care Team has enough time for me when I talk to them* |
| *Condition is controlled enough to let me go on with daily life / usual activities* | 120 | | Not included |
| *Have had a good experience of care in the past year* | 120 | Reword as "I had a good experience of care in the past year" | Not included |
| *Acceptability of appointment waiting time* | 120 | | *The lead time for getting appointments scheduled is acceptable* |
| *The doctors do not always know my medical history and problems very well* | 110 | Reword positively "The doctors always know my medical history and problems" | **I find that important information about me and my situation reaches the various people who need it: a) The hospital Care Team (for Crohn's and Colitis); b) Teams in other hospital departments that I may be in contact with; c) My primary care team (e.g. at my GP Practice)* |
| *Enough time to cover everything during appointments* | 110 | | **I feel that the Care Team has enough time for me when I talk to them* |
| *Personal circumstances and preferences considered* | 100 | | **I have the confidence to express my personal needs and concerns with members of the Care Team* |

| | | | |
|---|---|---|---|
| *Given understandable information* | 100 | Discussed importance of focussing on 'understandable to me'. | *About the information I was given: a) It was given in a way that was easy to understand* |
| *Given enough information* | 90 | Reword as "I was given enough information for my needs" or "I was given all the information I needed" | *About the information I was given: b) It was sufficient for my needs* |
| *Did you receive information regarding problems to be expected in the future?* | 90 | Discussed adding 'might experience' or 'potential problems'. | *About the information I was given: d) It helped me understand possible issues that might be expected in the future related to my Crohn's or Colitis* |
| *Good understanding of the treatment I am on or offered?* | 90 | Reword as "I have a good understanding of the treatment I am offered and/or the treatment I am on" | *I know what care and treatment options are available for my Crohn's or Colitis (including any alternatives to my current treatment)* |
| *Able to access different health team members* | 80 | Reword as "I can easily access my hospital team" or "I find it easy to be able to access different health team members" | **It is easy to get the help I need from a member of the Care Team when I need it* |
| *Feel able to approach member of health team to discuss any worries about treatment or its effect on my life* | 80 | Reword as "I feel able to approach the team to discuss any concerns about treatment or its effects on my life" | *I feel able to approach the Care Team to discuss any concerns about treatment or its effects on my life* |
| *The doctors know about non-medical things in my life (family, job, hobbies, social life)* | 70 | Combine with "*The team respect me as a fully-rounded person, not defining me by my medical situation"* | **My Care Team understands what's important to me as an individual (my preferences and priorities in healthcare and beyond)* |

| | | | |
|---|---|---|---|
| *Took your concerns seriously* | 70 | Reword as "Feeling cared for" or "The doctors listen to my concerns and take them seriously" | *My concerns are taken seriously when I talk to members of the Care Team* |
| *The team respect me as a fully-rounded person, not defining me by my medical situation* | 60 | Combine with "The doctors know about non-medical things in my life (family, job, hobbies, social life)" | **My Care Team understands what's important to me as an individual (my preferences and priorities in healthcare and beyond)* |
| *Treated respectfully as an individual* | 60 | | *I am treated respectfully as an individual by members of the Care Team* |
| *I don't always feel comfortable answering the doctors questions* | 50 | Reword positively "I feel I can discuss any concerns with my medical team" | **I have the confidence to express my personal needs and concerns with members of the Care Team* |
| *Did you experience that important information about you and your situation reached the staff who needed it?* | 50 | | **I find that important information about me and my situation reaches the various people who need it: a) The hospital Care Team (for Crohn's and Colitis); b) Teams in other hospital departments that I may be in contact with; c) My primary care team (e.g. at my GP Practice)* |
| *I was given information in a format that helped me understand the benefits and risks of surgery* | 50 | Discussed making more generic rather than focussing on surgery as this is not relevant to all. | *About the information I was given: e) The information was provided in a format I was happy with (i.e. whether it was given verbally or in writing)* |
| *The doctors I see in clinic communicate well with each other* | 40 | | **I find that important information about me and my situation reaches the various people who need it: a) The hospital Care Team (for Crohn's and Colitis); b) Teams in other hospital departments that I may* |

| | | | |
|---|---|---|---|
| | | | *be in contact with; c) My primary care team (e.g. at my GP Practice)* |
| *I feel that staff do their best to give me the care I need* | 40 | | *I feel that members of the Care Team do their best to give me the care I need* |
| *Did you feel that the doctors had enough time for you when you needed it?* | 30 | Reword as "Doctors have enough time for me when I need it " or "When meeting members of my health team I am given enough time to talk to them about what matters to me" | **I feel that the Care Team has enough time for me when I talk to them* |
| *Feel able to discuss personal/intimate issues with the team if I want to* | 30 | | *I feel able to discuss my mental health with the Care Team if I want to _(rest covered by individual needs and concerns)* |
| *The doctors know how I feel emotionally while they are treating me* | 30 | Discussed including signposting to emotional/psychological support, and to reword to "The doctors care how I feel emotionally while they are treating me" | *The Care Team knows how I feel emotionally while they are treating me* |
| *There is good co-ordination between the different health professionals in my care and treatment* | 20 | Combine with other items: "My care is well-coordinated between my GP and Gastroenterologist (e.g. blood tests, monitoring drug levels)" | **There is good coordination between the different health professionals involved in my care and treatment (e.g. my hospital Care Team, teams in other hospital departments, my GP, other health professionals)* |
| *It is very important to me to see my regular doctor* | 20 | Reword to include medical team rather than doctor. | *I usually see the same person (e.g. doctor or nurse) from the Care Team at each appointment.* |

| | | | |
|---|---|---|---|
| *Made aware of health team looking after me* | 20 | Reword as "I know who makes up the health team looking after me" | *I was made aware of who the various people are in the Care Team looking after me* |
| *Did the staff talk to you so that you understood what they meant?* | 20 | | *The way that members of the Care Team talk to me helps me understand the information they are giving me* |
| *I want one doctor to coordinate all the care I receive* | 10 | Include "in relation to my Ulcerative Colitis / Crohn's Disease" | *I know who on the Care Team is responsible for co-ordinating my care* |
| New suggestion | *N/A* | Should I need to, I know how to contact the IBD team between appointments. | *I know how to contact the Care Team between appointments if I need to* |
| New suggestion | *N/A* | Did you feel like it was easy to get help from a member of the IBD team when needed? | **It is easy to get the help I need from a member of the Care Team when I need it* |
| New suggestion | *N/A* | Are you able to discuss other issues related to the condition with the team (e.g. mental health, sexual function if stoma, etc.)? | *I feel able to discuss my mental health with the Care Team if I want to* |
| New suggestion | *N/A* | Do you find yourself updating people you see with information you feel they should already have? | Not included |

** = agreed Item covers two or more candidate Items*

**Supplementary Material 3. Items reworded to reduce the Gunning Fog Index Readability Formula Score.**

| Item | Score[1] | Revised item | Score[1] |
|---|---|---|---|
| *I know what care and treatment options are available for my Crohn's or Colitis, including any alternatives to my current treatment* | 12 | *I know what care and treatment options are available for* my *Crohn's and Colitis* | 8 |
| *The information was provided in a format I was happy with* | 8 | *I liked the way it was given (e.g. verbal or on paper)* | 5 |
| *I was made aware of who the various people are in the Care Team looking after me* | 9 | *I know who the different people are in the Care Team looking after me* | 8 |
| *I am able to get a response from the Care Team by the end of the next working day, when I need to* | 9 | *I can get a response from the Care Team by the end of the next working day* | 7 |
| *I feel able to approach the Care Team to discuss any concerns about treatment and its effects on my life* | 8 | *I feel I can approach the Care Team to discuss any concerns about treatment and its effects on my life* | 8 |
| *The Care Team ask how I feel emotionally while they are treating me* | 8 | *The Care Team ask how I feel while they are treating me* | 5 |
| *I receive enough social support from the people around me (friends, family or workplace) to help me live with Crohn's or Colitis.* | 12 | *I get enough support from the people around me to help me live with Crohn's or Colitis (such as friends, family or work)* | 6 |
| *I am treated respectfully as an individual by the Care Team* | 12 | *I am treated with dignity and respect by the Care Team* | 8 |
| *My Care Team understands what's important to me as an individual (my preferences and priorities in healthcare and* | 18 | *The Care Team understands what matters to me (in healthcare and beyond)* | 8 |

| | | | |
|---|---|---|---|
| *beyond)* | | | |
| *I have the confidence to express my individual needs and concerns with the Care Team* | 11 | *I have the confidence to express my needs and concerns with the Care Team* | 8 |
| *Over the last two weeks, I feel that I can cope and live with Crohn's or Colitis on a day to day basis* | 9 | *In general, I feel that I can cope mentally with my Crohn's or Colitis* | 11 |
| *I know the person or health professional on the Care Team who is responsible for coordinating my care and Treatment* | 14 | *I know the person on the Care Team who coordinates my care* | 8 |
| *There is good coordination between the different health professionals involved in my care and treatment* | 14 | *There is good coordination between the different people involved in my care and treatment* | 11 |
| *I can do all of the tasks that my care team have asked me to do at home (e.g. manage my diet, lifestyle changes, take medication)* | 8 | *I can do all of the tasks that my Care Team ask me to do at home* <br> *(such as such as manage my diet, lifestyle, and treatment)* | 7 |
| *On an ongoing basis I know how to monitor my symptoms* | 8 | *I am able to keep track of my symptoms* | 4 |
| *Overall, I believe that my care and treatment plan will benefit me* | 11 | *I believe that my care and treatment will benefit me* | 8 |

*Note: The word 'Colitis' was considered a 'complex word' and therefore not included in the formula*
*[1]Scores were rounded up to the nearest integer.*

**Supplementary Material 4. Issues highlighted in the Think Aloud Interviews (Stage 2) and the changes agreed by *the expert group* in Workshop 3 (Stage 3)**

| Item | Suggested issues | Changes agreed |
|---|---|---|
| *I have a clear picture of where I want to be in terms of my Crohn's or Colitis* | IBD is too unpredictable to have 'clear goals'. | Decided by voting (Appendix 5). |
| *My mental health and well-being affect my ability to live with my Crohn's or Colitis* | Ambiguous, negatively worded, and overlaps with Item below. | Remove Item. |
| *I feel that I have the emotional strength to live with IBD on a day to day basis* | Emotional strength fluctuates, and the term is too strong/emotive. | Decided by voting (Appendix 5). |
| 3 items including *'members of the care team'* | The answer may depend on who in the team this refers to. | Remove the words '*members of the*'. |
| *About the information I was given…* | Ambiguous timing/occasion of when information was given. | Reword to '*Last time I was given information by my Care Team about my care and treatment…'* |
| *About the information I was given: it was sufficient for my needs* | Unclear what would be classed as 'sufficient' and how a patient should know this. | Reword to '*it met my needs*'. |
| *About the information I was given: it helped me to understand possible issues that might be expected in the future in relation to my Crohn's or Colitis* | Disagreement about whether being informed about the future is a positive thing. | Decided by voting (Appendix 5). |
| *I am able to quickly contact different members of the Care Team when I need to* | The perception of 'quickly' may differ between patients, and this can depend on whether the situation is an emergency. | Reword to '*I can get a response from the Care Team by the end of the next working day*'. |

| | | |
|---|---|---|
| *It is easy to get the help I need from a member of the Care Team when I need it* | Overlaps with Item above. | Remove Item. |
| *I know how to contact the Care Team between appointments if I need to* | This can depend on whether the situation is an emergency. | Keep Item the same. |
| *I understand how Crohn's or Colitis is affecting my body* | IBD affects more than just the body. | Reword to: '*I understand how Crohn's or Colitis affects me physically*'. |
| *The lead time for getting appointments scheduled is acceptable* | This may differ for routine or urgent appointments. | Keep Item the same. |
| *I am able to access sufficient support from the wider IBD community to help me live with Crohn's or Colitis* | This could put pressure on individuals to be accessing the community. Also, 'wider IBD community' is ambiguous. | Reword to: '*I can access support from the wider IBD community to help me live with Crohn's or Colitis if I want to (such as charities, online groups, support groups)*'. |
| *The Care Team are fully up to date with my medical situation* | This could be due to patients not informing the medical team. | Remove Item. |
| *On an ongoing basis I am able to monitor my disease activity* | The symptoms do not always reflect disease activity. | Reword to: '*I am able to keep track of my symptoms'*. |
| *I am treated respectfully as an individual by members of the Care Team* | Similar to Item '*My Care Team understands what's important to me as an individual'*. | Remove the term '*individual*' from first item and keep both Items. |
| *My concerns are taken seriously when I talk to members of the Care Team* | Similar to Item above. | Keep Item. |
| *I feel able to discuss my mental health with the Care Team if I want to* | Similar to Item '*I have the confidence to express my needs and concerns with members of the Care Team*' | Keep both Items. |

| | | |
|---|---|---|
| *There is good coordination between the different health professionals involved in my care and treatment (e.g. my hospital Care Team, other hospital teams, my GP, other health professionals)* | May not be relevant to all people, as some have little contact with other departments. | Reword to specify: '*There is good coordination between the people involved in my care: a) The Care Team (for Crohn's and Colitis) and other hospital teams I may be in contact with; b) My Care Team and primary care (e.g. at my GP Practice)*'. |
| *I find that important information about me and my situation reaches the various people who need it* | Similar to Item above. | Keep both Items. |
| *10 new items suggested* | Content validity – participants identified areas that were not covered. | Decided by voting (Appendix 5). |

**Supplementary Material 5. Voting responses ((Rows 1-5, n=4; Rows 6-10, n=5) of *the expert group* to changes suggested to the PREM based on the Think Aloud Interviews, and changes agreed in Workshop 4 (Stage 3).**

| Suggested change | Participant votes | | | Final decision |
|---|---|---|---|---|
| | Agree (%) | Not sure (%) | Disagree (%) | |
| Replace "*I have a clear picture of where I want to be in terms of my Crohn's or Colitis*" with "*I have a personalised written care plan*" | 100 | 0 | 0 | *I have a personalised written care plan* (plus definition of personalised written care plan) |
| Replace "*The last time I was given information by the Care Team about my care and treatment: d) it helped me understand possible issues that might be expected in the future*" | | | | *The last time I was given information by the Care Team about my care and treatment: d) I had the opportunity to ask questions about the future* |
| with "*It helped me know what the future might hold for me"* | 25 | 0 | 0 | |
| or "*I had the opportunity to ask questions about the future*" | 50 | 25 | 0 | |
| Replace "*I feel I have the emotional strength to live with IBD on a day to day basis*" with "*In general, I feel that I can cope mentally with my Crohn's or Colitis*" | 100 | 0 | 0 | *In general, I feel that I can mentally cope with my Crohn's or Colitis* |
| Remove item "*I find that important information about me and my situation reaches the various people who need it*" | 100 | 0 | 0 | Remove item |
| Replace "*I understand how Crohn's or Colitis affects my body*" with "*I understand how Crohn's or Colitis affects me physically*" | 75 | 25 | 0 | *I understand how Crohn's or Colitis affects me physically* |

| | | | | |
|---|---|---|---|---|
| Add new item: "*I am able to easily access toilet facilities at the hospital*" | 40 | 60 | 0 | *I am able to easily access toilet facilities at the hospital* |
| Add new item: "*The Care Team know how to refer me to other services if needed, such as mental health services*" | 80 | 20 | 0 | *The Care Team will refer me to other services if needed (mental health services, for example)* |
| Add new item: "*The Care Team is sufficiently staffed and have the capacity to deliver my care and treatment*" | 40 | 20 | 40 | Do not include |
| Add new item: "*The Care Team informs me about opportunities to take part in research studies and clinical trials*" | 40 | 40 | 20 | *The Care Team informs me about opportunities to take part in research studies and clinical trials* |
| Add new item: "*The Care Team have given me the option to have appointments in a format that works for me, such as face to face, by telephone or online*" | 60 | 40 | 0 | *The Care Team offers me appointments in a format that suits me, such as face to face, by telephone or video call* |
| Add new item: "*I know how to make a formal complaint about the service, should I need to*" | 60 | 20 | 20 | *I know how to provide feedback on the service, should I want to* |
| Add new item: "*I have been recommended or directed to good, reliable information resources, such as Crohn's & Colitis UK and the NHS website*" | 40 | 20 | 40 | *The Care Team has recommended or directed me to good, reliable information resources, such as Crohn's & Colitis UK and the NHS website* |
| Add new item: "*The Care Team offers holistic care whilst treating me, including emotional support*" | 60 | 0 | 20 | Do not include |
| Add new item: "*The frequency of my routine appointments is acceptable*" | 80 | 0 | 20 | The frequency of my routine appointments is acceptable |

| | | | | |
|---|---|---|---|---|
| Add new ‘*Sexual implications of IBD’* item – no suggested wording. | 20 | 20 | 20 | Do not include |

**Supplementary Material 6. PREM mapped to conceptual experience domains and Theoretical Domains Framework**[1]

| Item no. | Item | Experience Domain | TDF/COM-B domain |
|---|---|---|---|
| *The Care Team* | | | |
| **1** | I know who the different people are in the Care Team looking after me | Communication | Psychological Capability/ Knowledge |
| **2** | I know how to contact the Care Team between appointments if I need to | Communication | Psychological Capability/ Knowledge |
| **3** | I can get a response from the Care Team by the end of the next working day when experiencing a flare | Accessibility | Physical Opportunity/ Environmental context and resources |
| **4** | I feel that the Care Team has enough time for me when I talk to them | Communication | Physical Opportunity/ Environmental context and resources |
| **5** | I know the person on the Care Team who coordinates my care | Integration | Knowledge/ Psychological Capability |
| *What Matters to Me* | | | |
| **6** | The Care Team understands the impact my Crohn's or Colitis has on my life | Patient-Centred Care | Social Opportunity/ Social Influences and Support |

| | | | |
|---|---|---|---|
| **7** | My concerns are taken seriously when I talk to the Care Team | Patient-Centred Care | Reflective Motivation/ Social Role and Identity |
| **8** | The Care Team ask how I feel while they are treating me | Patient-Centred Care | Social Opportunity/ Social Influences and Support |
| **9** | I feel I can approach the Care Team to discuss any concerns about my treatment and its effects on my life | Patient-Centred Care | Reflective Motivation/ Beliefs about Capabilities |
| **10** | The Care Team understands what matters to me (in healthcare and beyond) | Patient-Centred Care | Reflective Motivation/ Social Role and Identity |
| **11** | I have the confidence to express my needs and concerns with the Care Team | Patient-Centred Care | Reflective Motivation/ Beliefs about Capabilities |
| **12** | I feel that the Care Team do their best to give me the care I need | Quality | Reflective Motivation/ Optimism |
| **13** | I am involved in decisions about my care and treatment | Involvement | Reflective Motivation/ Social Role and Identity |
| **14** | I feel I have a good relationship with my Care Team | - | Reflective Motivation/ Goals |
| **15** | I am treated with dignity and respect by the Care Team | Patient-Centred Care | Reflective Motivation/ Social Role and Identity |
| **16** | I usually see the same person from the Care Team at each appointment (either face-to-face, telephone or online). | Integration | Social Opportunity/ Social Influences and Support |
| **17** | The Care Team offers me appointments in a format that suits me, such as face to face, by telephone or video call | Accessibility | Physical Opportunity/ Environmental |

| | | | |
|---|---|---|---|
| | | | context and resources |
| **18** | There is good coordination between the different people involved in my care and treatment:<br><br>a) Within my Care Team (doctors, IBD nurse specialists, surgeons, dietitians, for example) | Integration | Physical Opportunity/ Environmental context and resources |
| | b) Between my Care Team and other teams in the hospital that I may be in contact with (rheumatology, dermatology, obstetrics, for example) | | |
| | c) Between my Care Team and my GP Practice | | |
| | d) Between my Care Team and other healthcare professionals | | |
| **19** | The Care Team will refer me to other services if needed (mental health services, for example) | Integration | Physical Opportunity/ Environmental context and resources |
| **20** | In general, I am able to understand all the information the Care Team gives me | Communication | Psychological Capability/ Knowledge |
| **21** | Thinking about the last time I was given information by the Care Team about my care and treatment:<br>a) It was given in a way that was easy to understand | Communication | Psychological Capability/ Knowledge |
| | b) It met my needs | | |
| | c) It was relevant to me and my needs | | |

| | | | |
|---|---|---|---|
| | d) I had the opportunity to discuss and ask questions about it | | |
| | e) I liked the way it was given (e.g. verbal or on paper) | | |
| **22** | The Care Team has recommended or directed me to good, reliable information resources, such as charities and the NHS website | Communication | Physical Opportunity/ Environmental context and resources |
| **23** | The Care Team informs me about opportunities to take part in research studies and clinical trials | Communication | Psychological Capability/Knowledge |
| **24** | The frequency of my routine appointments is acceptable | Accessibility | Physical Opportunity/ Environmental context and resources |
| **25** | I am able to easily access toilet facilities at the hospital | Environment and Facilities | Physical Opportunity/ Environmental context and resources |
| **26** | I know how to provide feedback on the service, should I want to | Communication | Psychological Capability/ Knowledge |
| *Living with Crohn's or Colitis* | | | |
| **27** | I know what care and treatment options are available for *my* Crohn's or Colitis | Communication | Psychological Capability/ Knowledge |
| **28** | I understand how Crohn's or Colitis affects me physically | Communication | Psychological Capability/ Knowledge |

| | | | |
|---|---|---|---|
| **29** | In general, I feel that I can mentally cope with my Crohn's or Colitis | Patient-Centred Care | Automatic Motivation/Emotion |
| **30** | I feel able to discuss my mental health with the Care Team if I want to | Patient-Centred Care | Reflective Motivation/ Beliefs about Capabilities |
| **31** | I *can do* all the tasks that my care team ask me to do at home (such as manage my diet, lifestyle, treatment) | - | Physical Capability/ Skills |
| **32** | I *remember to do* all of the tasks that my care team ask me to do (such as take tablets, keep a food diary, etc.) | - | Physical Capability/ Skills |
| **33** | I am able to keep track of my symptoms | - | Physical Capability/ Skills |
| **34** | I feel it is important to take an active role in my own healthcare | - | Reflective Motivation/ Social Role and Identity |
| **35** | I get enough support from the people around me to help me live with Crohn's or Colitis (such as friends, family or people at work) | Patient-Centred Care | Social Opportunity/ Social Influences and Support |
| **36** | I can access support from the IBD community to help me live with Crohn's or Colitis, if I want to (such as charities, online groups, support groups) | Patient-Centred Care | Social Opportunity/ Social Influences and Support |
| **37** | I believe that my care and treatment will benefit me | - | Reflective Motivation/ Beliefs about Consequences |
| **38** | I have a personalised written care plan* | - | Reflective Motivation/ Goals |

[1] Michie S. Applying and Building Theory for Behaviour Change TIDieR : Template for Intervention Description. Published online 2014:1-19.

**Supplementary Material 7. Workshop 3 Presentation slides to the expert group**

AWARE IBD

# Patient-Reported Experience Measure

# Since last time...

18 think aloud interviews

12 females, 6 males

Age range 26 - 82 years

16 White British, 1 Black Caribbean, 1 Asian Tamil

Employment: Full-time, part-time, self-employed and retired

Education Level: GCSE to postgraduate

14 Crohn's Disease, 4 Ulcerative Colitis

Locations: Sheffield, Doncaster, Barnsley, Derbyshire, Leeds, London, Kent, South East, Durham, Milton Keynes, Bristol, Scotland

# Today’s Workshop

1. Face validity
2. Item themes
   - Recurring themes
   - Overlapping items
1. Length
2. Content validity: New items
3. Comprehension
4. Layout and Structure

Note: there are minor changes and suggestions which are not covered in these slides, due to time constraints. These changes will be reflected in the final PREM version, which will be circulated after the workshop today.

# Face Validity

*“I liked the fact that you did ask about mental health, feeling able to discuss it with them. And also about the mental health and wellbeing, and emotional strength, I think that’s really good because sometimes that gets left out and it is a really important part of having a long term condition. So I was really pleased to see those in there”*

*“I thought it was great. I mean it’s got the most applicable questions out of any like data input I’ve been asked to do on my condition in the last 8, 9 years I’ve been living with it. Like this feels like it’s been designed to someone like me as opposed to sitting down with your GP and going so how are you feeling?”*

*“I don’t think I’ve ever been asked how I find my care and I find it gives you a chance to almost, if you’re not happy with it then you can talk about it a bit. Which I think is quite good that you get given that opportunity to go much more into detail. So I would be happy to fill it in and I think equally if you’re really happy about the service then you’d be happy to go into much more detail about why.”*

*“This questionnaire is kind of making me realise that the care I’ve had hasn’t been brilliant, really.”*

*“I think you’ve got everything in there”*

*“I think it’s quite comprehensive and yeah it’s good”*

*“I think it was a really good questionnaire”*

*“I think it gives a good general overview of living with Colitis, without going into too much detail”*

*“I think it was easy to understand, and encapsulated the whole service”*

*“I think it’s very good”*

# Item themes

# Item 34: ‘I have a clear picture of where I want to be in terms of my Crohn’s or Colitis

- Domain: Reflective Motivation, Goals

## IBD is too unpredictable to have goals, the future is unknown (9/18)

*“you just never know because all of a sudden you can have a flare out of absolutely nowhere so it’s hard to have a clear picture”*

*“I don’t think a Crohn’s and Colitis journey is a clear one, for anyone”*

*“the short term and long term are so kind of vast and unknown”*

*“You can’t really have a picture of where you want to be with your Crohn’s other than you just want to be better ... And when you are better you don’t wanna be poorly again. That’s, to me, the only picture of Crohn’s disease”*

1. Remove the question
   Or
2. Clarify the question e.g. “I know what I want to achieve in managing my Crohn’s or Colitis”

# Item 20: My mental health and well-being affect my ability to live with my Crohn’s or Colitis

- Domain: Patient-centred care, Emotion

## Unclear what the question is asking (5/18)

*“Not really sure what that one’s asking me”*

*“See you’ve got mental health and then you’ve got wellbeing, are you on about physical wellbeing or are you sort of referring to the mental wellbeing?”*

*“does this mean if you have an existing mental health condition/ problems, this impacts your ability to live well with your condition? Or do we mean My Crohn's and Colitis has an impact on my mental health and well-being? Or both”*

## Negative scoring (2/18)

*“it was at the wrong end of the scale, you expect the 5s to be the positives and the 1s to be the negatives, whereas that one was switched round”*

## Overlap with Item 21: “I feel that I have the emotional strength to live with IBD on a day to day basis”

## Solution: Remove item 20

# Item 21: I feel that I have the emotional strength to live with IBD on a day to day basis

- Domain: Patient-centred care, Emotion

## Emotional strength fluctuates (5/18)

- Should responses reflect how the participant feels on that day, that week, or in general?

*“That varies, obviously depending on flare ups and different things. It can be as low as a 2 I’d say, but can also be a 4”*

*“if you’re talking about right now, this minute as I answer the question?”*

*“I don’t know what you mean by emotionally? At that very time, about the Crohn’s, or just generally in life?”*

- Emotional strength is too strong / emotive (if answering 1, not at all, implies weakness)
- Not understandable to younger service users 16-25
- ‘Coping’ and ‘ability’ as preferred terms

Solution: Over the last 2 weeks, I feel that I can cope and live with Crohn’s or Colitis

Item 7: I feel that the Care Team has enough time for me when I talk to them

Item 8: I am able to quickly contact different members of the Care Team when I need to

Item 11: I feel able to approach the Care Team to discuss any concerns about treatment or its effects on my life

Item 12: The Care Team knows how I feel emotionally while they are treating me

Item 16: My concerns are taken seriously when I talk to members of the Care Team

Item 17: My Care Team understands what's important to me as an individual (my preferences and priorities in healthcare and beyond)

Item 18: I feel able to discuss my mental health with the Care Team if I want to

Item 24: I usually see the same person e.g. doctor or nurse from the care team at each appointment

## Depends *who* on the care team (6/18)

*"It really does depend on who you speak to. Speak to a gastroenterologist; they normally understand a lot more than the IBD nurse"*

## Solutions:

1. Remove 'members of the' from Items 7, 8, 16
2. Remove item 24

# Item 2 ‘About the Information I was given…’

- Domain: Communication, Psychological Cap, Knowledge

## Timing/Occasion of when information was given (6/18)

- At diagnosis?
- Information given most recently? (e.g. about a treatment)
- In general?

*“I probably would have put don’t know, because I can’t remember the information I was given”*

*“it was 7 years ago now that I was diagnosed, erm, so I can’t remember what I was given and in what format, or what was said in the appointment”*

*“ A lot of the information that I’ve received from my care team hasn’t been for some time, but I’m trying to think back to when I did receive some”*

*“ it depends what it is and when it is”*

Solution: ‘The last time I was given information about my care and treatment…’

# Item 2b About the Information I was given: it was sufficient for my needs

- Domain: Communication, Psychological Cap, Knowledge

What is ‘sufficient’? How do I know this? (2/18)

*“I suppose it depends what you mean by sufficient?”*

*“I mean ‘sufficient for my needs’, what’s that trying to ask me?”*

- Would a patient necessarily know if information was sufficient for their needs or not?

Solution:  It met my needs

## Item 2d About the Information I was given: it helped me to understand possible issues that might be expected in the future in relation to my Crohn's or Colitis

- Domain: Communication, Psychological Cap, Knowledge

- Not necessarily a good thing
  - Patients may not want to know their prognosis, potential issues with treatments, possibility of surgery
- Inappropriate for newly diagnosed or younger patients, may feel overwhelming
- Gunning Fog index score of 18!!

Solution: Remove?

# Item 8 I am able to quickly contact different members of the Care Team when I need to

- Domain: Communication, Physical Opportunity, Environmental context and resources

People's perception of 'quickly' will differ (2/18)

- 24 hours? Within a working day?

*"I suppose it depends what, what the idea of quickly is. My idea of quickly might not be the same as someone else's"*

*"Maybe timely"*

Solution: I can get a response from the care team by the end of the next working day

Item 5 I know how to contact the Care Team between appointments if I need to

- Domain: Communication, Psychological Cap, Knowledge

Item 8 I am able to quickly contact different members of the Care Team when I need to

- Domain: Communication, Physical Opportunity, Environmental context and resources

Depends on the situation (emergencies yes, non-emergencies no) (1/18)

*“I got given a number to call if I needed to you know make an emergency appointment […] but I could never contact someone to just discuss something. [...] So I know how to contact someone in an emergency but not necessarily if anything non-emergency happens”*

*“Again in an emergence yes in a non-emergency no”*

Solutions:

1. Leave 5 as is
2. Reworded Item 8: I can get a response from the care team by the end of the next working day
3. Reorder the items together (item 8 becomes item 6)
4. Any other suggestions?

# Item 6 I understand how Crohn's or Colitis is affecting my body

- Domain: Communication, Psychological Cap, Knowledge

## Affects more than just the body (3/18)

*"So Crohn's isn't just about bowel disease to me, it's about my whole life, it affects me in so many ways"*

*"I think my question would be is that right now, or is that potential future effects as well?"*

Does this include mental health / the mind as part of the body?

Solutions:

1. Leave it as is
   Or
2. Rephrase: I understand how my Crohn's and Colitis affects my body (or gut?)
   And
3. Add a note to say we will address the emotional/psychological aspects later on in the questionnaire

## Item 10: The lead time for getting appointments scheduled is acceptable

- Domain: Accessibility, Physical Opportunity, Environment and resources

Different for routine vs. urgent appointments (1/18)

*“I wouldn’t have agreed with that at first because I was waiting a while even though I had an urgent referral […] But since then my appointments have been scheduled quite well”*

*“is that in relation to the previous questions, number 9 and number 8, how quickly I can get in touch with them? Or is that in relation to my annual, or however frequently the person you’re speaking to has an appointment? You might want to clarify number 10”*

‘Lead time’ considered a corporate word, comprehension issues

Solution: split into a,b
I am happy with the time it takes to schedule

a) Routine appointments
b) Urgent appointments

Item 14 I am able to access sufficient support from the wider IBD community to help me live with Crohn’s or Colitis

- Domain: Social opportunity, social support

- Need to define ‘wider community’ - e.g. within the hospital department, online, face-to-face (1/18)
- Puts pressure on individuals to be accessing the IBD community (1/18)

Solution: I can access support from the IBD community to help me live with Crohn’s or Colitis

## Item 23 The Care Team are fully up to date with my medical situation

- Domain: Quality, Reflective Motivation, Optimism

If not at all, is this because the patient hasn't informed them rather than issues with the Care Team?

What does this tell us about their experience?

Solution: Remove

## Item 30 On an ongoing basis I am able to monitor my disease activity

- Domain: Physical capability, Skills

Symptoms don’t reflect disease activity (3/18)

*“My symptoms don’t seem to reflect my disease activity”*

*“I don’t think it’s possible to monitor my disease activity, because I think the hidden activity is literally that, it’s hidden”*

*“my symptoms haven’t always typically matched my disease activity. Sometimes I’ve been feeling pretty good, but actually there’s a lot of inflammation going on.*

*“I’m wondering about whether the question should be ‘does the hospital care team help to monitor it actively?’”*

Solution: I know how to keep track of my symptoms

# Overlapping Items

Item 8 I am able to quickly contact different members of the Care Team when I need to

- Domain: Communication, Physical Opportunity, Environment and resources

Item 9  it is easy to get the help I need from a member of the Care Team when I need it

- Domain: Accessibility, Physical Opportunity, Environment and resources

## Overlap between 8 and 9 (2/18)

*“that’s quite similar to question 8, erm I’m having to kind of think about the difference between being able to quickly contact them, and getting the help I need. So I kind of feel like that’s part of the same question”*

*“ easy is, to me, the ease of ability to get hold of them is related to how long it takes, and how easy it is to get through, so really it’s the same as question 8. Question 8 and question 9, I’m taking to mean effectively the same thing”*

Note item 8 may have changed to “ I can get a response from the Care Team by the end of the next working day”

Item 15: I am treated respectfully as an individual by members of the Care Team

- Domain: Patient centred care, Social Opportunity, Social influences and support

Item 16: My concerns are taken seriously when I talk to members of the Care Team

Item 17: My Care Team understands what's important to me as an individual (my preferences and priorities in healthcare and beyond)

- Domain: Patient centred care, Reflective, Social role and identity

## Overlap between items 15, 16 and 17 (3/18)

*"Number 16, [...] I think I probably thought about that question whilst I was answering question 15. So maybe I would go back to question 15 and actually change that"*

*" 15 and 16 could probably be combined in some way"; "I do think number 15 and 17 are quite similar"*

*"Number 17[...] So I feel like that's a bit like number 15. [...] I think by having the word individual in there, as well as in the question number 17, I think that's what threw me a bit"*

Solution: 'I am treated with dignity and respect by the Care team"

Remove 17? Gunning Fog index score of 18

Item 18 I feel able to discuss my mental health with the Care Team if I want to

- Domain: Patient centred care, Reflective Motivation, Social Role and Identity

Item 19 I have the confidence to express my needs and concerns with members of the Care Team

- Domain: Patient centred care, Reflective Motivation, Beliefs about Capabilities

## Similar, could be combined? (2/18)

*“Question 18 and 19 could probably be combined”*

*“This is tied in, in my opinion this is really tied in to question 18”*

Item 26 There is good coordination between the different health professionals involved in my care and treatment (e.g. my hospital Care Team, other hospital teams, my GP, other health professionals)

- Domain: Integration, Social Opportunity, Social support

Item 27 find that important information about me and my situation reaches the various people who need it (a,b,c)

- Domain: Integration, Physical Opportunity, Environment and resources

## Similar items (2/18)

*"They read the same to me"; " Co-ordination for me is around sharing of information"*

## Problem of universality (5/18)

*"I've not really had much contact with other departments"*

Solution:

- Remove item 27
- Break down item 26 into a, b, c
- Combine a and b: my Care Team and other hospital teams

# Length

# Length

*“I think you probably wouldn’t want it to be too much longer, because I know people do get put off. But as it is, I think that’s fine”*

*“I think the balance is probably okay there. If it starts to take much longer than an hour I think you might find people struggle for time. But no I think, I think you’ve got a good balance there”*

*“I think even if there was a few more that you wanted to put in I think that would be fine”*

*“I think it’s a perfectly acceptable amount and you could have more if you needed to”*

*“Yeah I would be [...] happy to do it. I think anything that could potentially improve care for myself and future people would be great”*

*“I think it was a reasonable number of questions”*

*“I think it’s a good length cause I think it gives you a real like quite in-depth insight”*

# Content Validity
# New Items

# Suggestions for new items

1. Facilities
   - *"I am able to easily access toilet facilities at the hospital"*
1. IBD-related comorbidities
   - *"My care team recognise symptoms of IBD-related comorbidities"*
   - *My care team knows how to signpost me to MH services*
1. Referrals to other services
   - *My care team are able to signpost and refer me to other services if needed (e.g. mental health services)*
1. IBD-team staffing capacity
   - *"The care team is sufficiently staffed and has the capacity to deliver my care and treatment"*
1. Research opportunities
   - *"I am informed about opportunities to take part in research studies and clinical trials"*
1. Appointment format
   - *"I have been given the option to have appointments face to face, by telephone or online"*
1. Making a complaint
   - *"I know how to make a formal complaint about the service, should I find the need to"*

# Suggestions for new items

1. Information provision
   - *“I have been recommended or directed to good, reliable information resources, such as CCUK, NHS website”*
1. Holistic care
   - *“The care team offers holistic care whilst treating me, including emotional and psychological support”*
1. Frequency of appointments
   - *“The frequency of my routine appointments is acceptable”*
      - *Covered by item 10?*
1. Sexual implications of IBD
   - ? *“One of the things that does crop up online is sexual implications of IBD. That seems to crop up a lot in those sort of things. And that’s something that you can seem to be able to get no response from departments now. So it may be worth a question there to see if it’s something you can get support on”*

# Comprehension

*“I think it was very clear. I straight away knew who that referred to. I think ‘care team’ is probably quite a good way of putting it”*

*“I think it’s easy to understand yeah”*

“*No I think it’s surprisingly good. I think I’d normally, normally there’s something ambiguous or too abstract in all things like this, but no I think generally it’s fairly well done, it all read very well*”

*“They were quite like, not basic but they’re quite understandable questions, easy to answer”*

# Care Team Definition (11/18)

Include IBD Nurse

Include GP

Some people don't have a full team, e.g. just see a specialist or IBD nurse
- Do we need to recognise this?

*"...it was a little bit confusing I thought maybe, I didn't think the GPs were part of the care team because they weren't listed at the top but where does the separation happen?"*

*"My GP does actually play quite an active role"*

*"I can imagine some people would be panicking saying what doctors, nurses, others, who are they, who are the others? That's my reaction. Are there any? Who else, who else should be involved that I don't know about because I'm not an expert in this area? So who are you referring to, that I don't know about, that maybe should be involved in my care?"*

*"Because I haven't actually been physically to the hospital with my IBD yet, because of obviously all the covid-19, so I've only had telephone calls with the consultant. So yeah you might want to think about that, also in relation to the fact that there might be some patients who never go into the hospital."*

*"So I'd reference maybe IBD nurse somewhere, because that might be the only person they see."*

# Layout and Structure

# Layout

- 1-5 scale needs to be visible whilst answering questions (10/18)
- Add a ‘thank you note’ (1/18)
- Care Team definition missed (8/18)
  - Make bold, different colour
  - Add prompts, e.g. a function where you can hover your mouse over ‘care team’ to see the definition

# Structure

Group questions by:

- Items related to the Care Team / Service
- Items related to the mental health and emotional wellbeing
- Items related to living with IBD

5/18

# What's next?

Feedback on minor changes via email

- See PREM version 5 tracked changes

Final version circulation

**Supplementary Material 8. Workshop 4 Presentation slides to the expert group**

AWARE IBD

# Patient-Reported Experience Measure

# Today’s workshop

1. Voting results
2. Outstanding items to resolve
3. New items (based on voting spreadsheet)
4. Comprehension feedback
    a. Care Team definition

# Voting results for revised items

| Original item | Revised item | Outcome | Comments |
|---|---|---|---|
| I have a clear picture of where I want to be in terms of my Crohn's or Colitis | I have a personalised written care plan* (including a definition - see notes) | 4 / 4 Agree | |
| The last time I was given information by the Care Team about my care and treatment, d) it helped me understand possible issues that might be expected in the future | I had the opportunity to ask questions about the future | 3 / 4 Agree<br>1 / 4 Not sure<br>Outcome: Agree | X2 agreed with 'I had the opportunity to ask questions about the future'<br>X1 agreed with 'It helped me understand what the future might hold for me' |
| I feel I have the emotional strength to live with IBD on a day to day basis | In general, I feel that I can cope mentally with my Crohn's or Colitis | 4 / 4 Agree | |
| I find that important information about me and my situation reaches the various people who need it | Remove item | 4 / 4 Agree | |
| I understand how Crohn's or Colitis affects my body | I understand how Crohn's or Colitis affects me physically | 3 / 4 Agree<br>1 / 4 Not sure<br>Outcome: Agree | Needs simplifying for reading level - but appropriate?<br><br>Suggested rewording: I understand what symptoms are caused by Crohn's or Colitis (physical and mental) |

# Outstanding items to resolve

# The lead time for getting appointment scheduled is acceptable

Points to consider:

1) Time taken to have an appointment made

Or

1) Time between appointments

E.g. doctor wants to see you in 3 months but there’s no appointments for 6 months

Routine appointments or urgent appointments?

Is ‘lead time’ clear to younger age groups, newly diagnosed etc?

Item 26 There is good coordination between the different health professionals involved in my care and treatment (e.g. my hospital Care Team, other hospital teams, my GP, other health professionals)

- Domain: Integration, Social Opportunity, Social support

Item 27 find that important information about me and my situation reaches the various people who need it (a,b,c)

- Domain: Integration, Physical Opportunity, Environment and resources

Similar items (2/18)

*“They read the same to me”; “ Co-ordination for me is around sharing of information”*

Problem of universality (5/18)

*“I’ve not really had much contact with other departments”*

Item 26 and 27 Gunning Fog indexes >8

Solutions:

- Remove item 27 - agreed
- Reword item 26: There is good coordination between the people involved in my care
- Break down item 26 into a, b, c
- Combine a and b (to overcome the problem of universality): my Care Team (for Crohn’s and Colitis) and other hospital teams

# New Items

# 1. Facilities

## ‘I am able to easily access toilet facilities at the hospital’

2 /5 Include

3 /5 Not sure

Outcome: Requires further discussion

Comments

“I find it hard to conceive of any hospital not having adequate toilet facilities. Not sure how helpful this question would be to the IBD team, in any event, because if it IS an issue it is a wider issue for hospital mgt and is definitely not about the service provided by the IBD team.”

# 2. Referrals to other services

## The Care Team knows how to refer me to other services if needed, such as mental health services

4 /5 Include

1 /5 Not Sure

Outcome: Include

Comments:

This question can really only be included if it has a "don't know" or "N/A" option. Many people will not have needed further referral and will not have any idea if the care team can do this. If no DK or N/A option is possible then exclude it.

"The Care Team refer me to other services if needed, such as mental health services" The care team will know how to - the issue whether they will.

# 3. IBD-Team capacity

## The Care Team is sufficiently staffed and have the capacity to deliver my care and treatment

2 / 5 Include
2 /5 Exclude
1 / 5 Not Sure

Outcome: requires further discussion

Comments

Staffing may be difficult to judge from the patient's point of view and may lead to patients answer they agree with the statement  because of long appointment times which may not be due to staffing (waiting times have already been covered on another question)

Suggested wording: The Care Team APPEARS TO BE sufficiently staffed and has the capacity to deliver my care and treatment. (Patients cannot really know whether it is sufficiently staffed or not, only how it appears to them. (And it is 'has' not 'have').

There is a risk that appointment lead time issue will automatically result in this Q being answered negatively. Not sure whether that is an issue for the research survey results' interpretation.

Suggested solution: "The care team appears to have the capacity to deliver my care and treatment"

# 4. Research opportunities

## The Care Team informs me about opportunities to take part in research studies and clinical trials

2 / 5 Include

1 / 5 Exclude

2 / 5 Not Sure

Outcome: Requires further discussion

Comments

Is this is applicable to all IBD centres? as I am not sure if all centres carry out studies/clinical trials.

Happy to see it included IF the local IBD team thinks this is something that they are concerned with. I'm not sure that (a) each hospital has that many research opportunities that it can involve its own patients in, and (b) that the local IBD team's role is to recruit for national/non-local-hospital-based research

# 5. Appointment format

## The Care Team has given me the option to have appointments in a format that works for me, such as face to face, by telephone or video call

3 /5 Include

2 /5 Not Sure

Outcome: Include

Comments

During Covid many people who may have wanted F2F appointments were denied them. This is still the case for many hospitals who are insisting on phone only. In the fullness of time this is a good question to add, but maybe not this year.

Not suitable for likert scale?

# 6. Making a complaint

## I know how to make a formal complaint about the service, should I need to

3 /5 Include

1 /5 Exclude

1 /5 Not Sure

Outcome: Include

Comments

Not an aspect of the actual IBD team's service delivery.

Not suitable for likert scale

# 7. Information Provision

## I have been recommended or directed to good, reliable information resources, such as Crohn's & Colitis UK and the NHS website

2 /5 Include

2 /5 Exclude

1 /5 Not Sure

Outcome: requires further discussion

Comments

Needs to be made clear this comes from the care team

# 8. Holistic Care

## The Care Team offers holistic care whilst treating me, including emotional support

3 /5 Include

2 /5 Exclude

Outcome: Include

Comments

Potentially already covered in one of the other questions.

Include, in principle, but need to check overlap with other Qs

- Note: potential overlap with “The Care Team understands what matters to me (in healthcare and beyond)” and “I feel able to discuss my mental  health with the Care Team if I want to”

Does everyone understand holistic?

# 9. Frequency of appointments
## The frequency of my routine appointments is acceptable

4 /5 Include

1 /5 Exclude

Outcome: Include

Comments

Could replace Q6: “The lead time for getting appointments scheduled is acceptable”

# 10. Sexual implications of IBD

1 /3 Include
1 /3 Exclude
1 /3 Not Sure

Outcome: requires further discussion

Comments

If this gets a lot of 'to some extent' or more responses, it may signal to the IBD team that they need to include sexual health within their day-to-day conversations with patients. On the other hand, it may turn out to be a non-issue.

Sexual health is not within the remit of an IBD team. I suspect some respondents would prefer not to answer any such question. And if it is about 'can you get support for sexual issues' again it must have a DK or N/A option (or a 'you can leave this blank' flag) as many will not have had any need, so will have no opinion or experience with which to respond.

The wording of a slightly different question, might help the IBD team understand the level of hidden demand though.

Solutions: "My Crohn’s or Colitis has a negative impact on my sexual health or sex life"
or
"If I felt my sex life was being affected by my having Crohn’s or Colitis, I am confident that the care team would be able to signpost me to appropriate support."

# 11. The Care Team understands the impact my Crohn's or Colitis has on my life

Suggested new item by email

Comments

In relation to item "I feel I can approach the Care Team to discuss any concerns about my treatment and its effects on my life"

Note that this is about the effects on my life of the treatment and any concerns re that. The bigger question: The Care Team understands the impact of MY Crohn's or Colitis on my life seems to be unasked.

# Comprehension

# Comprehension

*“I think it was very clear. I straight away knew who that referred to. I think ‘care team’ is probably quite a good way of putting it”*

*“I think it’s easy to understand yeah”*

“*No I think it’s surprisingly good. I think I’d normally, normally there’s something ambiguous or too abstract in all things like this, but no I think generally it’s fairly well done, it all read very well*”

*“They were quite like, not basic but they’re quite understandable questions, easy to answer”*

# Care Team Definition (11/18)

Include IBD Nurse

Include GP

Some people don't have a full team, e.g. just see a specialist or IBD nurse
- Do we need to recognise this?

*"...it was a little bit confusing I thought maybe, I didn't think the GPs were part of the care team because they weren't listed at the top but where does the separation happen?"*

*"My GP does actually play quite an active role"*

*"I can imagine some people would be panicking saying what doctors, nurses, others, who are they, who are the others? That's my reaction. Are there any? Who else, who else should be involved that I don't know about because I'm not an expert in this area? So who are you referring to, that I don't know about, that maybe should be involved in my care?"*

*"Because I haven't actually been physically to the hospital with my IBD yet, because of obviously all the covid-19, so I've only had telephone calls with the consultant. So yeah you might want to think about that, also in relation to the fact that there might be some patients who never go into the hospital."*

*"So I'd reference maybe IBD nurse somewhere, because that might be the only person they see."*

# What’s next?

Revise the PREM to reflect new decisions

Circulate PREM to the group via email

Write up the paper for publication

- Opportunity to be involved
- Co-authorship

Newsletter

# Thank you!

**Supplementary File 9. Ad hoc voting by *the expert group* on solutions and suggested new items where required by Stage 3 interview findings**

*Please indicate your vote by typing an X in the relevant column (Agree / Disagree / Not sure). Any items without a clear majority vote or with a majority vote of 'Not sure' will be discussed at the workshop. You can also use the final column to suggest how to improve the wording of the item, if needed.*

| Original item | Solution | Agree | Disagree | Not sure | If you would like to reword the item, please type your suggestions below: |
|---|---|---|---|---|---|
| I have a clear picture of where I want to be in terms of my Crohn's or Colitis | I have a personalised written care plan*<br><br>*This is different to a first treatment plan. A care plan is a document that sets out how your Crohn's or Colitis will be treated, managed and monitored. It should take into consideration your hopes and life aspirations. It is an agreement or plan between you and your healthcare professional to help meet those goals. This is different to the letters or correspondence that a doctor may send you or your GP after each appointment. | XXXX | | | |
| The last time I was given information by the Care Team about my care and treatment:<br>d) it helped me understand possible issues that | It helped me know what the future might hold for me<br>or<br>I had the opportunity to ask questions about the future | XXX | | X | It helped me to find out what the future might hold for me<br><br>X2 Agree for I had the opportunity to ask questions... |

| | | | | |
|---|---|---|---|---|
| might be expected in the future | | | | |
| I feel I have the emotional strength to live with IBD on a day to day basis | In general, I feel that I can cope mentally with my Crohn's or Colitis* | XXXX | | I feel that I can cope mentally with my Crohn's or Colitis<br><br>I feel I have the mental strength to live with my Crohn's or Colitis |
| I find that important information about me and my situation reaches the various people who need it | Remove item | XXXX | | |
| I understand how Crohn's or Colitis affects my body | I understand how Crohn's or Colitis affects me physically<br><br>(Needs simplifying, readability score too high) | XXX | X | I understand how my Crohn's or Colitis affects my physical health.<br><br>I understand what symptoms are caused by Crohn's or Colitis (physical and mental). |
